# The Relationship between Lipoprotein A and the Prevalence of Multivessel Coronary Artery Disease in Young Patients with Acute Myocardial Infarction: An Observational Study

**DOI:** 10.3390/biomedicines12092159

**Published:** 2024-09-23

**Authors:** Ionut Cezar Buciu, Eugen Nicolae Tieranu, Andreea Stefania Pircalabu, Ovidiu Mircea Zlatian, Ionut Donoiu, Constantin Militaru, Sebastian Militaru, Cristian Militaru

**Affiliations:** 1 Doctoral School, University of Medicine and Pharmacy of Craiova, 200349 Craiova, Romania; cezarbuciu@yahoo.com; 2 Department of Cardiology, University of Medicine and Pharmacy of Craiova, 200349 Craiova, Romania; eugen.tieranu@umfcv.ro (E.N.T.); ionut.donoiu@umfcv.ro (I.D.); constantin.militaru@umfcv.ro (C.M.); crmilitaru@gmail.com (C.M.); 3 Department of Cardiology, Craiova Emergency Clinical County Hospital, 200642 Craiova, Romania; 4 Department of Oncology, Craiova Emergency Clinical County Hospital, 200642 Craiova, Romania; pircalabuandreea@yahoo.com; 5 Department of Microbiology, University of Medicine and Pharmacy of Craiova, 200349 Craiova, Romania; ovidiu.zlatian@umfcv.ro; 6 Medical Laboratory, Craiova Emergency Clinical County Hospital, 200642 Craiova, Romania; 7 Cardiomed Hospital, 200032 Craiova, Romania

**Keywords:** lipoprotein A, myocardial infarction, coronary lesions, cardiovascular disease, cardiovascular risk

## Abstract

**Introduction:** Cardiovascular diseases are the leading cause of mortality worldwide, with a significant impact on socioeconomic aspects. Various biomarkers have been studied in relation to the diagnosis, progression, and prognosis of atherosclerotic disease, with lipoprotein (a) [Lp (a)] standing out as an important predictor of cardiovascular risk. This observational study aimed to clarify the association between Lp (a) levels and the severity of significant multivessel coronary lesions in acute myocardial infarction (AMI) patients. **Materials and Methods:** Conducted at the Clinical Emergency County Hospital of Craiova, Romania, the study involved 256 young patients divided into two groups based on Lp (a) levels: Group A (Lp (a) < 30 mg/dL) and Group B (Lp (a) ≥ 30 mg/dL). Patients included young adults up to 55 years for males and 60 years for females, excluding those with familial hypercholesterolemia. **Results:** The study revealed a significant association between elevated Lp (a) levels and the presence of multivessel coronary lesions. Patients with Lp (a) concentrations ≥ 30 mg/dL exhibited a higher prevalence of multivessel disease compared to those with lower levels. **Discussion:** The findings suggest that elevated Lp (a) levels are a crucial biomarker for the risk of coronary artery disease, particularly in young patients with AMI. The study emphasizes the need for aggressive lipid management strategies and personalized treatment approaches, considering the significant role of Lp (a) in atherosclerosis and AMI. **Conclusions:** Lipoprotein A levels above 30 mg/dL are associated with a higher prevalence of multivessel coronary lesions. Multivariate analysis revealed that higher Lp (a) levels and lower HDL levels are linked to an increased risk of multivessel coronary lesions.

## 1. Introduction

Cardiovascular diseases (CVDs) are the leading cause of mortality worldwide and also have a major impact on socioeconomic aspects. Among the numerous manifestations of CVD, acute myocardial infarction (AMI) is of particular concern because of its high morbidity and mortality rates. Acute myocardial infarction (AMI), represented by ST-segment elevation myocardial infarction (STEMI) and non-ST-segment elevation myocardial infarction (NSTEMI), poses a major issue to the healthcare system because of its increasing rates of morbidity and mortality. Biomarkers play pivotal roles in the diagnosis, prognosis, and management of these conditions. While various biomarkers, such as lipoprotein (a) [Lp (a)], have been extensively studied in the context of predicting cardiovascular events, including AMI, and the severity of coronary heart disease, the novelty of Lp (a) as a biomarker has been challenged by a growing body of literature. The differential impact of high versus normal Lp (a) levels on the risk and outcomes of patients with acute myocardial infarction is not fully understood, with existing studies offering inconclusive or contradictory results [1,2,3,4].

Lp (a) is no longer considered a novel marker for coronary artery disease (CAD) or AMI, with multiple studies associating elevated Lp (a) levels with atherosclerosis, atherothrombosis, and plaque instability [5]. Indeed, Lp (a) has been shown to be associated with LDL-C and involved in the risk of cardiovascular events [6,7]. Currently, there is a growing body of clear evidence indicating the role of Lp (a) in the processes of inflammation, the formation and maintenance of atheroma plaques, and intra-arterial thrombosis through oxidized phospholipids [8]. These processes lead to the onset of ischemic cardiovascular disease and its most severe complication, myocardial infarction [1,9].

However, despite advances in our understanding of biomarkers related to single-vessel coronary disease and AMI, the identification of biomarkers specifically predictive of multivessel disease (MVD) remains underexplored. MVD, which involves significant stenosis in more than one coronary artery, is associated with worse clinical outcomes and higher mortality rates than single-vessel disease. Thus, the need for more targeted biomarkers to predict and stratify risk in patients with MVD is paramount.

In this context, our observational study aimed to elucidate the relationship between Lp (a) concentration and the occurrence of multivessel coronary lesions in young patients admitted with acute myocardial infarction. Conducted at the Clinical Emergency County Hospital of Craiova, Romania, this study involved 256 patients categorized into two groups based on their Lp (a) levels: Group A with Lp (a) < 30 mg/dL and Group B with Lp (a) ≥ 30 mg/dL. This division allows for comparative analysis to evaluate the role of high Lp (a) levels as a potential independent risk factor for more severe coronary involvement in patients with acute myocardial infarction. The results of this study could provide valuable insights into the pathophysiological mechanisms linking Lp (a) to adverse cardiovascular outcomes and could help refine risk stratification strategies for patients presenting with acute myocardial infarction.

## 2. Materials and Methods

Building upon the foundational premises previously articulated, this study embarked on a prospective investigation at a single center located in Romania, within the European Union. A cohort of 256 consecutive patients, admitted to the Emergency Department of the Clinical County Emergency Hospital of Craiova and diagnosed with acute myocardial infarction of both STEMI and NSTEMI types, according to the fourth definition of acute myocardial infarction, was enrolled in 2022. The selection criteria included adults aged up to 55 years for males and 60 years for females, who underwent angiographic examination within the first 12 h of myocardial infarction onset by interventional cardiologists, excluding individuals previously diagnosed with familial hypercholesterolemia or those who died before discharge.

Patient demographic information, such as sex, age, area of residence, overweight status, smoking habits, and diabetes status, was systematically collected from the hospital’s electronic records. Upon admission to the Cardiology Unit, all participants underwent clinical examination by a cardiologist within the first two hours. Diagnostic procedures included a 12-lead electrocardiogram displaying typical myocardial infarction modifications, conducted using a GE Healthcare MAC2000 (New York, NY, USA), and transthoracic echocardiography performed using a GE Healthcare Vivid E90 (Chicago, IL, USA). Biochemical parameters were analyzed in the hospital laboratory, and lipid profiles and lipoprotein (a) levels were assessed using the COBAS INTEGRA 400 PLUS system (Roche, Basel, Switzerland), employing the Tina-quant Lipoprotein (a) (Latex) test for lipoprotein (a) concentration measurements.

All patients underwent emergency coronary angiography that was performed in the hospital’s angiography and cardiac catheterization laboratory by interventional cardiologists. Independent of the chosen therapeutic approach, these specialists established the angiographic diagnosis of the lesions by analyzing the images. Lesions were considered significant if they involved an endoluminal narrowing of more than 50% in the left main artery and at the origin of the anterior descending, circumflex, and right coronary arteries, and >70% throughout the remaining coronary distribution. Furthermore, patients diagnosed with lesions in more than one coronary artery were classified under the category of multivessel disease.

Statistical analysis was performed using STATA 17 SE (StataCorp LLC, College Station, TX, USA) to exclude patients with nonsignificant coronary stenosis from the risk factor analysis associated with multivascular lesions. Based on lipoprotein (a) levels, the patients were classified into Group A (lipoprotein (a) < 30 mg/dL; 128 patients) and Group B (lipoprotein (a) ≥ 30 mg/dL; 128 patients). This division was predicated on the existing literature suggesting that lipoprotein (a) levels ≥ 30 mg/dL significantly increase the risk of major adverse cardiovascular events in patients with myocardial infarction [10] and exacerbate the severity of coronary atherosclerosis in younger populations [11].

Continuous variables were expressed as the median and interquartile range (IQR) and compared using the Kruskal–Wallis test. Categorical variables were presented as frequencies and percentages and compared using the chi-square test or Fisher’s exact test where appropriate. The relative risk (RR) with 95% confidence intervals (CIs) was calculated.

A multivariate logistic regression model was used to identify independent predictors of multivessel coronary lesions. Variables with a *p*-value < 0.05 in univariate analysis were included in the model. Odds ratios (ORs) with 95% CIs were reported. The Hosmer–Lemeshow test was used to assess the goodness-of-fit of the model.

A *p*-value < 0.05 was considered statistically significant for all tests. Data analysis was performed adhering to the intention-to-treat principle to account for all participants.

## 3. Results

**Figure 1:**
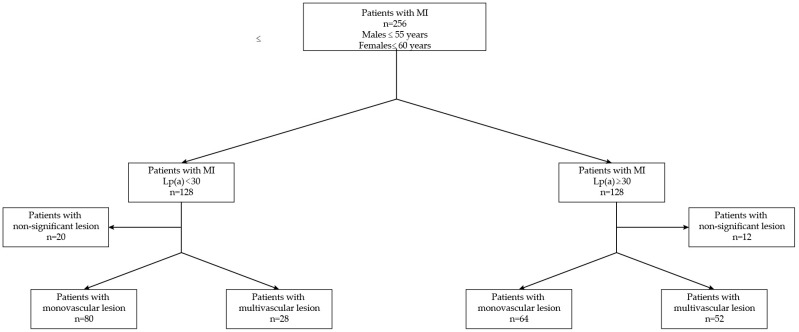
Stratification of 256 patients with myocardial infarction (MI) based on lipoprotein (a) [Lp (a)] levels and angiographic diagnosis.

Figure 1 illustrates the stratification of 256 patients with myocardial infarction (MI) divided into two groups based on lipoprotein (a) [Lp (a)] levels. Males aged ≤ 55 years and females aged ≤ 60 years were included in the study. Patients were divided into two groups: those with Lp (a) levels < 30 (*n* = 128) and those with Lp (a) levels > 30 (*n* = 128).

Each group was further subdivided based on the severity of the coronary artery lesions. In the Lp (a) < 30 group, 20 patients had non-significant lesions, 80 had monovascular lesions, and 28 had multivascular lesions. In the Lp (a) > 30 group, 12 patients presented with nonsignificant lesions, 64 with monovascular lesions, and 52 with multivascular lesions.

**Figure 2:**
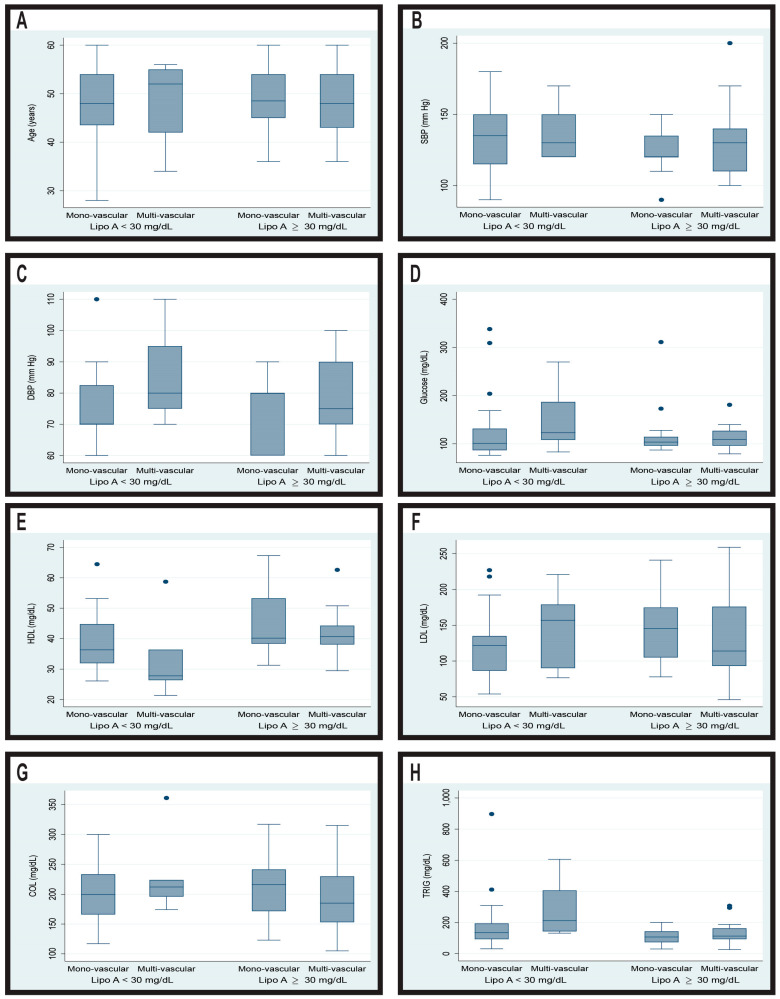
Variation of biological parameters between patients with monovascular lesions and multivascular lesions according to Lipo(a) levels. SBP: systolic blood pressure; DBP: diastolic blood pressure; HDL: high-density lipoprotein cholesterol; LDL: low-density lipoprotein cholesterol; COL: total cholesterol; TRIG: triglycerides. (**A**) age; (**B**) systolic blood pressure; (**C**) diastolic blood pressure; (**D**) glucose; (**E**) HDL cholesterol; (**F**) LDL cholesterol; (**G**) total cholesterol; (**H**) triglycerides.

Table 1 and Figure 2 present the baseline characteristics of the 256 patients enrolled in the study who were diagnosed with acute coronary syndrome. The analysis excluded patients with nonsignificant coronary lesions to focus on the risk factors associated with multivascular lesions.

The majority of the patients were male (79.69%, *n* = 204), which is consistent with broader cardiovascular epidemiological trends that show a higher prevalence of acute myocardial infarction (AMI) among men. Only 20.31% (*n* = 52) of patients were female. Regarding geographical distribution, 59.38% (*n* = 152) of the patients lived in urban areas, while 40.63% (*n* = 104) lived in rural regions. This reflects potential differences in lifestyle factors, such as diet and activity levels, which contribute to the risk of cardiovascular disease.

Obesity and overweight were common among the study participants, with 39.06% (*n* = 100) classified as obese and 34.38% (*n* = 88) classified as overweight. A smaller proportion, 26.56% (*n* = 68), had a normal weight. Smoking was identified as a major risk factor, with 66.02% (*n* = 169) of the patients being active smokers, emphasizing the importance of smoking cessation programs as part of cardiovascular disease prevention strategies and reducing the incidence of myocardial infarction. Diabetes mellitus and hypertension (HBP) were present in 28.13% (*n* = 72) and 67.58% (*n* = 173) of the patients, respectively. Both are well-known risk factors for myocardial infarction and unfavorable cardiovascular outcomes. Dyslipidemia was the most frequent condition, observed in 88.67% (*n* = 227) of patients, highlighting its significant role in the pathophysiology of AMI.

Typical anginal chest pain was the most common presenting symptom, reported by 79.69% (*n* = 204) of patients, while 20.31% (*n* = 52) experienced atypical chest pain. The median age of the patients was 48.5 years (IQR: 43.5–54 years), highlighting the relatively young age of this group of patients with acute coronary events. The median systolic blood pressure (SBP) was 130 mm Hg (IQR: 120–145 mm Hg), and the median diastolic blood pressure (DBP) was 80 mm Hg (IQR: 70–90 mm Hg), reflecting moderate blood pressure levels within the group.

Coronary angiographic findings showed that 56.25% (*n* = 144) of the patients had monovascular disease, while 31.25% (*n* = 80) were diagnosed with multivascular lesions, indicating varying degrees of coronary artery disease severity.

The lipid profile showed suboptimal levels of high-density lipoprotein (HDL), with a median value of 39.64 mg/dL (IQR: 32.67–45.45 mg/dL), further emphasizing the high cardiovascular risk. Low-density lipoprotein (LDL) cholesterol had a median value of 126.3 mg/dL (IQR: 90.2–162.13 mg/dL), while total cholesterol had a median value of 205 mg/dL (IQR: 167.5–235 mg/dL). Triglycerides showed considerable variability, with a median value of 130.5 mg/dL (IQR: 87.5–190.5 mg/dL). Glycemic control was generally adequate, with a median glucose level of 108.5 mg/dL (IQR: 94–129 mg/dL) and a median HbA1c level of 5.74% (IQR: 5.38–6.45%), although some patients exhibited pre-diabetic or poorly controlled diabetes markers.

### 3.1. Comparison of Risk Factors and Biologic Parameters between Patients with Lipoprotein A Levels < 30 mg/dL and ≥30 mg/dL

As shown in Table 2, we conducted a comparative analysis of patient parameters with lipoprotein A values below 30 ng/mL compared to those with lipoprotein A values equal to or exceeding 30 mg/dL.

The divergences observed in the demographic variables (Table 2) illustrate a higher proportion of males in both groups (87.50% in Group A, 71.88% in Group B), with a statistically significant difference between the groups (RR = 0.82, *p* = 0.002). The median age in Group A (median: 50.5 years, IQR: 44–54.5) was marginally elevated in comparison to Group B (median: 48 years, IQR: 43–53.5), although this divergence was not statistically significant (*p* = 0.203). Concerning area of residence, no noteworthy difference was distinguishable between urban and rural residents across groups (*p* = 0.309); however, the majority of patients were living in urban areas (59.38%).

The prevalence of overweight and obese conditions showed pronounced variance between the groups, exhibiting a lower propensity in Group A comparative to Group B (15.62% overweight individuals in group A vs. 37.50% overweight individuals in Group B; *p* < 0.001). The incidence of smoking was virtually identical in both groups, with no significant difference (*p* = 0.692).

Diabetes had a slightly lower prevalence in Group B (25.00%) than in Group A (31.25%), although this difference was not statistically significant (*p* = 0.266). Dyslipidemia showed high incidences across both groups, with no significant divergence discerned (*p* = 0.844). The prevalence of high blood pressure was similar between the two groups, demonstrating no significant variation (*p* = 0.689).

Regarding symptom onset, no considerable distinction was discernible between the groups in terms of pain characterization (atypical vs. typical pain) (*p* = 0.5343). Nonetheless, a marked divergence was evident in the lesion types, with a higher prevalence of multivascular lesions in Group B (44.83%) than in Group A (25.93%; translating into a relative risk (RR) of 1.73 (*p* = 0.003)). 

The systolic blood pressure was significantly augmented (*p* < 0.001) in Group A (median: 142.5 mm Hg, IQR: 120–150) relative to Group B (median: 125 mm Hg, IQR: 120–140). Diastolic blood pressure values were not significantly different between the groups (*p* = 0.070).

As expected, differences were observed in the lipid metrics between the groups. HDL cholesterol readings were significantly reduced in Group A (median: 36.36 mg/dL, IQR: 30.37–44.8) compared to Group B (median: 40.19 mg/dL, IQR: 37.88–50.35; *p* < 0.001). LDL cholesterol values were marginally reduced (*p* = 0.050) in Group A, presenting a median of 126.3 mg/dL (IQR: 88.345–153) compared to 125.65 mg/dL (IQR: 98.5–171.5) in Group B. Neither total cholesterol (COL) levels nor glucose concentrations showed significant differences between groups (*p* = 0.344). Triglyceride levels, however, were significantly amplified (*p* < 0.001) in Group A (median: 148.5 mg/dL, IQR: 112–250.5) compared to Group B (median: 110.5 mg/dL, IQR:78.5–159; *p* < 0.001).

Glucose levels did not differ significantly between the groups, with Group A (lipoprotein A < 30 mg/dL) demonstrating a median glucose quantification of 114 mg/dL (interquartile range [IQR]: 89.5–172) and Group B (lipoprotein A ≥ 30 mg/dL) presenting a marginally reduced median of 105.5 mg/dL (IQR: 96.5–122.5). Despite the seemingly reduced median glucose levels in Group B, this variance was not statistically significant (*p* = 0.344). Moreover, glycated hemoglobin (HbA1c) concentrations also manifested significant contrasts (*p* = 0.042), with Group A revealing a median of 5.75% (IQR: 5.44–7.86) compared to 5.59% (IQR: 5.34–6.34) in Group B (Table 2).

### 3.2. Risk Factors and Biological Parameters for Multivascular vs. Monovascular Lesions in Patients with Lipoprotein A Levels < 30 mg/dL

Our data (Table 3) demonstrated a significantly pronounced dissimilarity in the sex of the patients between the monovascular and multivascular lesion groups. Male patients accounted for 85.00% of the monovascular group, while they encompassed 100.00% of the multivascular group, yielding a statistically relevant risk for males (RR = 1.17, *p* = 0.030). The median age, however, did not show substantial variation between the two groups (*p* = 0.367). The patient residence area also did not demonstrate significant differences between the two lesion categories (*p* = 0.281).

Being overweight emerged as a consequential risk factor for multivascular lesions (RR = 1.33, *p* = 0.001), with 42.86% of patients identified as overweight relative to 45.00% in the monovascular group. The multivascular lesion cohort displayed a higher percentage of smokers (RR = 2.30, 82.14% vs. 61.25%, *p* = 0.043).

We observed a prominent divergence in the prevalence of diabetes between the two groups. A remarkable percentage (71.43%) of patients with multivascular lesions were diagnosed with diabetes compared to a mere 20.00% in the monovascular category, confirming a significant association (RR = 5.00, *p* < 0.001). Dyslipidemia prevalence was comparably high across both groups without significant alterations (*p* = 0.536).

Systolic and diastolic blood pressure measurements were relatively similar; however, diastolic pressure presented a significant difference (*p* = 0.003) and was elevated in the group with multivascular lesions. Paradoxically, hypertension (HBP) was more prevalent in the monovascular group (75.00% vs. 42.86%, with RR = 0.38, *p* = 0.002). Symptom onset presented a significant difference; 100.00% of the multivascular group complained of typical pain, in contrast to 80.00% in the monovascular group (RR = 0.80, *p* = 0.010).

Regarding lipid profiles, HDL cholesterol levels were significantly lower in patients with multivascular lesions (median: 27.8 mg/dL, IQR: 26.4–36.43, *p* = 0.001), coupled with significantly inflated LDL cholesterol (median: 157 mg/dL, IQR: 90–179, *p* = 0.029). Interestingly, total cholesterol levels did not delineate any consequential variance between the groups (*p* = 0.144). Triglyceride levels were markedly elevated in the multivascular lesion group (median, 212 mg/dL; IQR: 143–407, *p* = 0.003). Glucose levels were also notably increased in the multivascular cluster (median: 123 mg/dL, IQR: 108–187) compared to the monovascular group (median: 101 mg/dL, IQR: 86.5–131.5, *p* = 0.018). Additionally, glycated hemoglobin (HbA1c) levels were significantly elevated in the multivascular group, indicating poorer long-term glucose management (median: 8.46%; IQR: 6.58–10.28, *p* < 0.001).

### 3.3. Risk Factors and Biological Parameters for Multivascular vs. Monovascular Lesions in Patients with Lipoprotein A Levels ≥ 30 mg/dL

Our study found no noteworthy differences concerning the distribution of gender (*p* = 0.956), age demographics (*p* = 0.593), or residence area (*p* = 0.078) (Table 4). However, a salient variation was discerned in the body mass indices of the patients, revealing a preponderance of overweight and obese individuals within the multivascular lesion group (RR = 0.49, *p* = 0.003). The prevalence of smoking was found to be elevated among the monovascular lesion patients (RR = 0.61, *p* = 0.018). Other parameters including residential locality, incidence of diabetes, dyslipidemia, and hypertension did not exhibit substantial variances between the two lesion categories.

Upon comparing the biochemical and clinical parameters between the two groups, no significant differences were uncovered in several areas. These include age, lipid profile (encompassing HDL, LDL, overall cholesterol, and triglycerides), as well as glucose and HbA1c levels, further emphasizing the complexity of these lesion subtypes.

### 3.4. Severity of Coronary Lesions Based on Lipoprotein (a) Levels in Major Coronary Arteries

**Figure 3:**
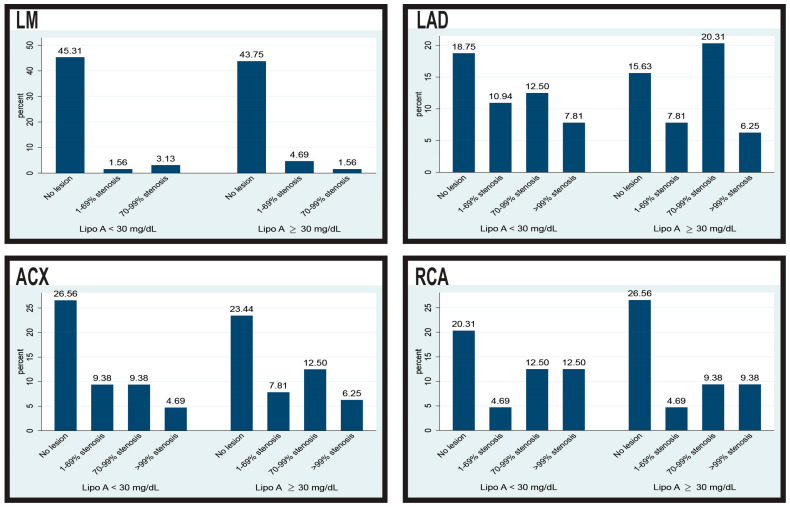
Severity of coronary lesions based on lipoprotein (a) levels in the major coronary arteries. LM: left main; LAD: left anterior descending artery; ACX: circumflex artery; RCA: right coronary artery.

In Figure 3, we assessed the severity of coronary artery lesions in the four major coronary arteries according to lipoprotein (a) [Lp (a)] levels, stratified into two categories: Lp (a) < 30 mg/dL and Lp (a) ≥ 30 mg/dL. For the left main artery, patients with Lp (a) < 30 mg/dL had slightly higher rates of no lesions (45.31% vs. 43.75%) and 70–99% stenosis (3.13% vs. 1.56%), whereas those with Lp (a) ≥ 30 mg/dL exhibited a higher prevalence of 1–69% stenosis (4.69% vs. 1.56%). In the left anterior descending artery (LAD), a greater proportion of patients with elevated Lp (a) had severe stenosis (20.31% had 70–99% stenosis compared with 12.50% in the lower Lp (a) group). Similarly, in the circumflex artery, patients with Lp (a) ≥ 30 mg/dL showed higher rates of 70–99% stenosis (12.50% vs. 9.38%) and >99% stenosis (6.25% vs. 4.69%). Lastly, for the right coronary artery (RCA), patients with higher Lp (a) had fewer severe lesions (9.38% had >99% stenosis compared to 12.50% in the lower Lp (a) group), although the rate of no lesions was higher in this group (26.56% vs. 20.31%). Overall, these findings suggest a trend toward more severe coronary lesions in patients with elevated Lp (a) levels, particularly in the LAD and circumflex arteries.

### 3.5. Multivariate Logistic Regression Analysis

We performed a multivariate logistic regression analysis (Table 5) to discern the association between the risk of multicoronary lesions and risk factors, excluding those presenting non-significant lesions.

The probability of being diagnosed with multicoronary lesions was markedly elevated for patients harboring increased lipoprotein A concentrations (odds ratio [OR]: 5.472, *p* < 0.001). Our study also unveiled a significant correlation between sex and the lesion category. Overweight male patients had significantly higher odds of developing multivascular disease (OR: 6.298, *p* = 0.050).

The likelihood of having multicoronary lesions was significantly higher in individuals with elevated lipoprotein A levels (OR: 5.472, *p* < 0.001). This implies that individuals presenting lipoprotein A levels ≥ 30 mg/dL have significantly amplified odds of having multicoronary lesions compared to those with lipoprotein A < 30 mg/dL. For each unit increment in HDL cholesterol, there was a concomitant decrease in the likelihood of a diagnosis with the multicoronary lesions (OR: 0.926, *p* < 0.001), implying that elevated HDL-C levels confer resilience against severe coronary lesions, consistent with other studies [12].

Our research also revealed a strong association between gender and lesion category. Among overweight male patients, there was a substantial likelihood of developing multivascular disease (OR: 6.298, *p* = 0.050). This suggests that overweight males are at a much higher risk of experiencing more severe forms of coronary artery disease, characterized by multivascular involvement, compared to their non-overweight or female counterparts. This finding is consistent with the broader understanding that body weight and sex are critical factors influencing cardiovascular risk profiles and disease severity.

A systematic review and meta-analysis by Mongraw-Chaffin et al. (2015) [13] found that the relationship between BMI and coronary heart disease (CHD) does not significantly differ between men and women. The study reviewed data from 95 cohorts involving 1.2 million participants and concluded that higher BMI is associated with an increased risk of CHD for both sexes. Specifically, a one-unit increase in BMI was linked to a similar increase in the hazard ratios for CHD in both women and men, indicating that the adverse effects of higher BMI on coronary heart disease risk are consistent across sexes.

Patients presenting hypertension exhibited diminished odds of being clinically classified with the target coronary lesion category (OR: 0.334, 95%, *p* < 0.001). This finding suggests a possible interplay between hypertension and the gravity of coronary lesions. Also, patients with diabetes had significant odds (OR: 6.263, *p* < 0.001) of having multicoronary lesions.

Additional parameters including comprehensive lipids, area of residence (urban versus rural), overweight status, smoking habits, and dyslipidemia were incorporated into the regression model. However, these variables did not achieve statistical relevance.

The logistic regression analysis underscores the significant impact of lipoprotein A levels, HDL, gender, and hypertension on the likelihood of being diagnosed with a particular category of coronary lesions in patients presenting with elevated lipoprotein A levels. These findings highlight the complex interplay of lipid profiles, demographic factors, and comorbid conditions in determining the severity of coronary artery disease, particularly in the context of acute myocardial infarction. Further research is warranted to elucidate the underlying mechanisms and potential clinical implications of these associations.

## 4. Discussion

The study results could be instrumental for tailoring interventions and understanding the lipidomic landscape in acute myocardial infarction patients. The findings suggest a need for aggressive lipid management strategies, potentially incorporating novel therapeutic agents that specifically target lipoprotein A or more aggressively manage LDL cholesterol, given their role in the progression of coronary artery disease.

The patients in our group were mostly under 55 years old (men) and under 60 years old (women); thus, the results demonstrate the involvement of Lipo (a) as a risk factor in the pathogenesis of MI in younger patients, as observed by Statescu et al. [14]. The study included only 52 female patients compared to 204 male patients, despite specifically aiming to evaluate the influence of gender. This disproportion represents a significant limitation of the research, as it reduces the statistical power of the findings due to the small number of female participants. Consequently, conclusions regarding the impact of gender should be interpreted with caution, as this imbalance in gender distribution may affect the validity and generalizability of the results.

Further, the variability in these parameters underscores the importance of personalized medicine approaches in the management of acute myocardial infarction, considering the wide range of values for key biomarkers like LDL, triglycerides, and glucose levels. In Table 2, we observe that while there is a trend towards lower glucose levels in patients with higher lipoprotein A levels, it may not be clinically significant within this population, as other studies suggest [15]. However, a study by Boronat et al. highlighted an inverse relationship between lipoprotein A levels and the prevalence of diabetes in older adults, suggesting a protective role of higher lipoprotein A levels against the occurrence of diabetes with advancing age [16]. The implications of these findings could suggest that lipoprotein A levels have a limited association with acute variations in glucose concentrations in patients presenting with acute myocardial infarction. However, the broader relationship between lipid metabolism and glucose regulation, especially in the context of cardiovascular diseases, remains an area requiring further exploration.

The study findings illustrate that elevated lipoprotein A levels are associated with certain cardiovascular risk factors such as systolic blood pressure, HDL, LDL, and triglycerides in patients presenting with acute myocardial infarction. Elevated lipoprotein A levels were associated with higher incidences of acute myocardial infarction, particularly in patients with normal levels of low-density lipoprotein cholesterol [10]. Moreover, within the high lipoprotein A cohort, the type of coronary lesion—monovascular vs. multivascular—appears to be influenced by body weight status and smoking prevalence, as demonstrated by studies on the Framingham Heart Offspring Cohort [17], but not other demographic, biochemical, or clinical parameters. These insights contribute to the understanding of the role of lipoprotein A in the context of coronary artery disease and its potential implications for patient management and prognosis.

Moreover, multivariate analysis confirmed that lipoprotein A levels were associated with a risk of multicoronary lesions. A study by Hoang et al. [18] found that patients with acute myocardial infarction and serum lipoprotein (a) ≥ 50 mg/dL had a higher rate of three-vessel disease, suggesting that high lipoprotein (a) levels may be associated with multicoronary lesions. Another study showed that patients with very high lipoprotein A levels (>80 mg/dL) exhibit a higher prevalence of a personal and family history of cardiovascular disease and are more frequently women. Based on coronary angiography results, these patients have an increased extent of coronary artery disease and more frequent multivessel disease, suggesting a link between elevated lipoprotein A levels and the severity of coronary disease in the context of MI [19].

Regarding the optimal cut-off for lipoprotein (a) levels for the risk of cardiovascular disease, a study on Lp (a) levels in patients with major cardiovascular events demonstrated a median value of 18 mg/dL [20]. According to results from the Cleveland Clinic, the patients with serum levels of Lp (a) higher than 31 mg/dL are considered at high risk for cardiovascular disease, and the highest risk is noted for those with levels above 50 mg/dL [21]. Although most studies use a cut-off of 50 mg/dL, we wanted to demonstrate that a lower cut-off of 30 mg/dL can better identify high-risk patients and support the relevance of our findings, highlighting the importance of considering lower limits in vascular risk assessment. 

The study’s finding that hypertensive patients showed a lower odds of being diagnosed with severe coronary lesions, despite the usual association between hypertension and cardiovascular risk, suggests that the aggressive management of hypertension with cardiovascular medications might have protective effects on the heart and blood vessels, reducing the severity of coronary lesions. This phenomenon could be attributed to early detection and intervention due to heightened medical surveillance and more comprehensive healthcare engagement among hypertensive individuals. The selection bias in the study sample might lead to underrepresentation of hypertensive patients with advanced coronary artery disease, as those with hypertension are more likely to receive timely medical attention and interventions, potentially preventing the progression to severe lesions. The interaction between blood pressure and coronary artery disease is multifaceted and shaped by various elements including blood pressure fluctuations and the performance of the endothelial layer. In certain instances, well-controlled hypertension may not impose identical shear stress on coronary arteries compared to uncontrolled or sporadic hypertension, potentially giving rise to divergent coronary artery lesion development patterns. As proposed by Cruickshank [22], individuals with pronounced stenosis of the coronary artery coupled with hypertension exhibit inadequate coronary flow reserve. This condition could render the myocardial tissue more susceptible to coronary perfusion pressures, which are otherwise well-tolerated in patients devoid of ischemia.

Lp (a) is a biomarker known for its stability over time, remaining constant under various physiological and pathological conditions, making it comparable to other markers used in forensic diagnostics. While previous studies have shown that LMAN2, CAPN-1, and VCP remain stable up to six hours postmortem and are not influenced by factors such as time of death, age, or postmortem interval, there are no studies specifically highlighting postmortem levels of Lp (a) [23].

Although our study focused on the role of Lp (a) in acute myocardial infarction, it is well known that its levels do not significantly change during acute conditions or after death. This stability provides Lp (a) with an important advantage in forensic medicine, where the reliability of serological markers is crucial for diagnosis. Therefore, Lp (a) could significantly contribute to the postmortem assessment of cardiac deaths and complement the current panel of biomarkers used in the diagnosis of EMI-SCD [24,25].

## 5. Conclusions

High plasma concentrations of lipoprotein (a) [Lp (a)] stand out as a significant biomarker for cardiovascular risk in the context of myocardial infarction. The study conducted at our center demonstrates that patients with Lp (a) levels above 30 mg/dL have a higher prevalence of multivessel coronary lesions compared to those with lower levels. This highlights the major role of Lp (a) in the development of atherosclerotic plaques and the severity of coronary artery disease.

Multivariate analysis revealed that higher Lp (a) levels and lower HDL levels are linked to an increased risk of multivessel coronary lesions, with an odds ratio of 5.472, reinforcing the necessity to consider Lp (a) levels in risk stratification and management strategies for AMI patients.

On the other hand, hypertension and diabetes have more complex relationships with the severity of coronary artery disease. The differential impact of high versus normal Lp (a) levels on cardiovascular outcomes emphasizes the need for personalized medicine approaches in managing AMI. The insights from this study advocate for incorporating Lp (a) level assessments in routine clinical evaluations to enhance the prognostic accuracy and therapeutic targeting in patients with acute myocardial infarction. This finding underscores the importance of including Lp (a) measurements in diagnostic strategies, recommending at least one lifetime measurement, as suggested by Kronenberg (2021) [26].

In conclusion, this study provides valuable insights into the mechanisms linking Lp (a) and the extent of coronary artery disease. This can help refine risk stratification strategies for patients with acute myocardial infarction. Further research is needed to understand the underlying mechanisms and establish the clinical implications of these associations.

## Figures and Tables

**Table 1:** Characteristics of the patients with acute coronary syndrome included in the study. HBP: high blood pressure. SBP: systolic blood pressure. DBP: diastolic blood pressure. HDL: high-density lipoprotein cholesterol. LDL: low-density lipoprotein cholesterol. COL: cholesterol. TRIG: triglycerides. HbA1C: hemoglobin A1c.

Discrete Variables		
Parameter	Total (*n* = 256)	
	No.	%
Sex		
Men	204	79.69%
Women	52	20.31%
Area of residence		
Urban	152	59.38%
Rural	104	40.63%
Overweight		
Normal weight	68	26.56%
Overweight	88	34.38%
Obesity	100	39.06%
Smoking		
Yes	169	66.02%
No	87	33.98%
Diabetes		
Yes	72	28.13%
No	184	71.87%
HBP		
Yes	173	67.58%
No	83	32.42%
Dyslipidemia		
Yes	227	88.67%
No	29	11.33%
Onset symptoms		
Atypical pain	52	20.31%
Typical pain	204	79.69%
Type of lesion		
Non-significant lesion	32	12.50%
Monovascular	144	56.25%
Multivascular	80	31.25%
**Continuous variables**		
**Parameter**	**Median**	**IQR**
Age (years)	48.5	43.5–54
SBP (mm Hg)	130	120–145
DBP (mm Hg)	80	70–90
Glucose (mg/dL)	108.5	94–129
HDL (mg/dL)	39.64	32.67–45.45
LDL (mg/dL)	126.3	90.2–162.13
COL (mg/dL)	205	167.5–235
TRIG (mg/dL)	130.5	87.5–190.5
HBA1c (%)	5.74	5.38–6.45

**Table 2:** Characteristics of the patients with Lipo A < 30 mg/dL compared with Lipo A ≥ 30 mg/dL. HBP: high blood pressure. SBP: systolic blood pressure. DBP: diastolic blood pressure. HDL: high-density lipoprotein cholesterol. LDL: low-density lipoprotein cholesterol. COL: cholesterol. TRIG: triglycerides. HbA1C: hemoglobin A1c.

Discrete Variables									
Parameter				Lipo A < 30 mg/dL *n* = 128		Lipo A ≥ 30 mg/dL *n* = 128		RR	*p*
				No.	%	No.	%		
Sex								0.82	0.002 *
Men				112	87.50%	92	71.88%		
Women				16	12.50%	36	28.12%		
Area of residence								0.90	0.309
Urban				80	62.50%	72	56.25%		
Rural				48	37.50%	56	43.75%		
Overweight								0.74	<0.001 *
Normal weight				20	15.62%	48	37.50		
Overweight				52	40.63%	36	28.12%		
Obesity				56	43.75%	44	34.38%		
Smoking								1.04	0.692
Yes				83	64.84%	86	67.19%		
No				45	35.16%	42	32.81%		
Diabetes								0.80	0.266
Yes				40	31.25%	32	25.00%		
No				88	68.75%	96	75.00%		
HBP								0.97	0.689
Yes				88	68.75%	85	66.41%		
No				40	31.25%	43	33.59%		
Dyslipidemia								1.01	0.844
Yes				113	88.28%	114	89.06%		
No				15	11.72%	14	10.94%		
Onset symptoms								1.17	0.534
Atypical pain				24	18.75%	28	21.88%		
Typical pain				104	81.25%	100	78.12%		
Type of lesion								1.73	0.003
Non-significant lesion				20	15.62%	12	9.37%		
Monovascular				80	62.50%	64	50.00%		
Multivascular				28	21.88%	52	40.63%		
**Continuous Variables**									
**Lipo A < 30 mg/dL** ***n* = 128**			**Lipo A ≥ 30 mg/dL** ***n* = 128**			***p***	**Total** ***n* = 256**		
Parameter	Median	IQR	Median		IQR		Median	IQR	
Age (years)	50.5	44–54.5	48		43–53.5	0.203	48.5	43.5–54	
SBP (mm Hg)	142.5	120–150	125		120–140	<0.001 **	130	120–145	
DBP (mm Hg)	80	70–90	80		67.5–85	0.070	80	70–90	
Glucose (mg/dL)	114	89.5–172	105.5		96.5–122.5	0.344	108.5	94–129	
HDL (mg/dL)	36.365	30.37–44.8	40.195		37.885–50.35	<0.001 **	39.64	32.67–45.45	
LDL (mg/dL)	126.3	88.345–153	125.65		98.5–171.5	0.050 **	126.3	90.2–162.13	
COL (mg/dL)	209.5	181.5–233.5	201		153.5–236	0.344	205	167.5–235	
TRIG (mg/dL)	148.5	112–250.5	110.5		78.5–159	<0.001 **	130.5	87.5–190.5	
HBA1c (%)	5.75	5.44–7.86	5.59		5.34–6.34	0.022 **	5.74	5.38–6.45	

*: *p* < 0.05 (chi-square test). **: *p* < 0.05 (Kruskal–Wallis test).

**Table 3:** Patient parameters according to coronary lesion type in patients with lipoprotein A levels < 30 mg/dL (patients with non-significant lesions were excluded). HBP: high blood pressure. SBP: systolic blood pressure. DBP: diastolic blood pressure. HDL: high-density lipoprotein cholesterol. LDL: low-density lipoprotein cholesterol. COL: cholesterol. TRIG: triglycerides. HbA1C: hemoglobin A1c.

Discrete Variables							
Parameter		Monovascular Lesion *n* = 80		Multivascular Lesion *n* = 28		RR	*p*
		No.	%	No.	%		
Sex						1.17	0.030 *
Men		68	85.00%	28	100.00%		
Women		12	15.00%	0	0.00%		
Area of residence						1.47	0.281
Urban		48	60.00%	20	71.43%		
Rural		32	40.00%	8	28.57%		
Overweight						1.33	0.001 *
Normal weight		8	10.00	12	42.86%		
Overweight		36	45.00%	4	14.28%		
Obesity		36	45.00%	12	42.86%		
Smoking						2.30	0.043 *
Yes		49	61.25%	23	82.14%		
No		31	38.75%	5	17.86%		
Diabetes						5.00	<0.001 *
Yes		16	20.00%	20	71.43		
No		64	80.00%	8	28.57%		
HBP						0.38	0.002 *
Yes		60	75.00%	12	42.86%		
No		20	25.00%	16	57.14%		
Dyslipidemia						1.47	0.536
Yes		71	88.75%	26	92.86%		
No		9	11.25%	2	7.14%		
Onset symptoms						0.80	0.010 *
Atypical pain		16	20.00%	0	0.00%		
Typical pain		64	80.00%	28	100.00%		
**Monovascular Lesion** ***n* = 80**			**Multivascular Lesion** ***n* = 28**		***p***	**Total** ***n* = 108**	
Parameter	Median	IQR	Median	IQR		Median	IQR
Age (years)	48	43.5–54	52	42–55	0.367	50.5	44–54.5
SBP (mm Hg)	135	115–150	130	120–150	0.734	142.5	120–150
DBP(mm Hg)	70	70–82.5	80	75–95	0.003 **	80	70–90
Glucose (mg/dL)	101	86.5–131.5	123	108–187	0.018 **	114	89.5–172
HDL (mg/dL)	36.33	31.94–44.8	27.8	26.4–36.43	0.001	36.36	30.37–44.8
LDL (mg/dL)	121.8	86.5–135	157	90–179	0.029	126.3	88.345–153
COL (mg/dL)	199.5	166–233.5	212	196–224	0.144	209.5	181.5–233.5
TRIG (mg/dL)	135	93–194.5	212	143–407	0.003 **	148.5	112–250.5
HBA1c (%)	5.73	5.38–6.07	8.46	6.58–10.28	<0.001 **	5.75	5.44–7.86

*: *p* < 0.05 (chi-square test). **: *p* < 0.05 (Kruskal-Wallis test).

**Table 4:** Patient parameters according to coronary lesion type in patients with lipoprotein A levels ≥ 30 mg/dL. (Patients with non-significant lesions were excluded). SBP: systolic blood pressure. DBP: diastolic blood pressure. HDL: high-density lipoprotein cholesterol. LDL: low-density lipoprotein cholesterol. COL: cholesterol. TRIG: triglycerides. HbA1C: hemoglobin A1c.

Monovascular Lesion				Multivascular Lesion		RR	*p*
Parameter		No.	%	No.	%		
Sex						1.01	0.956
Men		44	68.75%	36	69.23%		
Women		20	31.25%	16	30.77%		
Area of residence						0.70	0.078
Urban		40	62.50%	24	46.15%		
Rural		24	37.50%	28	53.85%		
Overweight						0.49	0.003 *
Normal weight		32	50.00%	12	23.08%		
Overweight		8	12.50%	24	46.15%		
Obesity		24	37.50%	16	30.77%		
Smoking						0.61	0.018 *
Yes		51	79.69%	31	59.62%		
No		13	20.31%	21	40.38%		
Diabetes						1.40	0.132
Yes		12	18.75%	16	30.77%		
No		52	81.25%	36	69.23%		
HTA						0.84	0.399
Yes		43	67.19%	31	59.62%		
No		21	32.81%	21	40.38%		
Dyslipidemia						0.60	0.060
Yes		60	93.75%	43	82.69%		
No		4	6.25%	9	17.31%		
Onset symptoms						1.15	0.567
Atypical pain		12	18.75%	12	23.08%		
Typical pain		52	81.25%	40	76.92%		
**Monovascular Lesion** ***n* = 64**			**Multivascular Lesion** ***n* = 52**		***p***	**Total** ***n* = 116**	
Parameter	Median	IQR	Median	IQR		Median	IQR
Age (years)	48.5	45–54	48	43–54	0.593	48.5	43.5–54
SBP (mm Hg)	120	120–135	130	110–140	0.684	130	120–145
DBP (mm Hg)	80	60–80	75	70–90	0.522	80	70–90
Glucose (mg/dL)	103.5	96–114.5	109	96–127	0.398	108.5	94–129
HDL (mg/dL)	40.19	38.37–53.26	40.7	38.1–44.3	0.230	39.64	32.67–45.45
LDL (mg/dL)	145.5	105–174.85	114	93–176	0.110	126.3	90.2–162.13
COL (mg/dL)	216	171.5–241.5	185	153–230	0.075	205	167.5–235
TRIG (mg/dL)	107	73–143.5	113	93–163	0.213	130.5	87.5–190.5
HBA1c (%)	5.49	5.31–6.24	5.78	5.36–6.32	0.658	5.74	5.38–6.45

* *p* < 0.05 (chi-square test). **: *p* < 0.05 (Kruskal-Wallis test).

**Table 5:** Multivariate logistic regression analysis for the odds of developing multicoronary lesions. HBP: high blood pressure. HDL: high-density lipoprotein cholesterol. LDL: low-density lipoprotein cholesterol.

Multicoronary Lesions	Odds Ratio	Std. Err.	Z	*p* > Z	[95% Conf. Interval]	
Lipoprotein A ≥ 30 mg/dL	5.472	2.197	4.23	<0.001 *	2.491	12.020
HDL cholesterol (mg/dL)	0.926	0.019	−3.75	<0.001 *	0.890	0.964
LDL cholesterol (mg/dL)	1.002	0.004	0.46	0.646	0.994	1.009
Urban area of residence	1.115	0.388	0.31	0.755	0.563	2.208
Smoking	1.021	0.382	0.06	0.956	0.491	2.125
HBP	0.334	0.128	−2.85	0.004 *	0.157	0.710
Dyslipidemia	0.390	0.239	−1.54	0.124	0.118	1.295
Diabetes	6.263	2.482	1.94	<0.001 *	2.880	13.619
Sex # Overweight						
Women # Overweight	1.490	1.535	0.39	0.699	0.198	11.231
Women # Obesity	2.511	2.702	0.86	0.392	0.305	20.691
Men # Not Overweight	2.053	1.852	0.8	0.425	0.351	12.029
Men # Overweight	6.298	5.930	1.95	0.050 *	0.995	39.855
Men # Obesity	1.863	1.738	0.67	0.505	0.299	11.591
Constant	6.180	7.463	1.51	0.132	0.579	65.910

*: *p* < 0.05; #: interaction term in regression equation.

## Data Availability

The data are available from the authors upon request.
